# From international health to global health: how to foster a better dialogue between empirical and normative disciplines

**DOI:** 10.1186/s12914-014-0036-5

**Published:** 2014-12-12

**Authors:** Gorik Ooms

**Affiliations:** Department of Public Health, Institute of Tropical Medicine, Nationalestraat 155, 2000 Antwerp, Belgium; Law and Development Research Group, Faculty of Law, University of Antwerp, Venusstraat 23, 2000 Antwerp, Belgium

**Keywords:** Global health, International health, Human rights, Ethics, Empirical, Normative, Is-ought problem, Interdisciplinary research

## Abstract

**Background:**

Public health recommendations are usually based on a mixture of empirical evidence and normative arguments: to argue that authorities ought to implement an intervention that has proven effective in improving people’s health requires a normative position confirming that the authorities are responsible for improving people’s health. While public health (at the national level) is based on a widely accepted normative starting point – namely, that it is the responsibility of the state to improve people’s health – there is no widely accepted normative starting point for international health or global health. As global health recommendations may vary depending on the normative starting point one uses, global health research requires a better dialogue between researchers who are trained in empirical disciplines and researchers who are trained in normative disciplines.

**Discussion:**

Global health researchers with a background in empirical disciplines seem reluctant to clarify the normative starting point they use, perhaps because normative statements cannot be derived directly from empirical evidence, or because there is a wide gap between present policies and the normative starting point they personally support. Global health researchers with a background in normative disciplines usually do not present their work in ways that help their colleagues with a background in empirical disciplines to distinguish between what is merely personal opinion and professional opinion based on rigorous normative research.

If global health researchers with a background in empirical disciplines clarified their normative starting point, their recommendations would become more useful for their colleagues with a background in normative disciplines. If global health researchers who focus on normative issues used adapted qualitative research guidelines to present their results, their findings would be more useful for their colleagues with a background in empirical disciplines.

**Summary:**

Although a single common paradigm for all scientific disciplines that contribute to global health research may not be possible or desirable, global health researchers with a background in empirical disciplines and global health researchers with a background in normative disciplines could present their ‘truths’ in ways that would improve dialogue. This paper calls for an exchange of views between global health researchers and editors of medical journals.

## Background

### Empirical versus normative disciplines and the ‘is-ought problem’

More than a century ago, Sabine wrote: “The general division of sciences into descriptive and normative has long been one of the commonest devices used in classification, so much that it has become almost traditional to refer to it even in the elementary text-books of logic and ethics” [1], page 433. Descriptive sciences, according to Sabine, “attempt merely to state *what is*” [1], page 433, emphasis in original. Normative sciences “are said to state not what is but *what ought to be*” [1], page 433, emphasis in original.

A century later, the division of sciences into descriptive and normative is no longer customary. We all understand that few sciences can be classified as purely descriptive or purely normative. Scientific work that attempts to understand *the world as it is* usually has a direct or indirect objective of improving the world, and is guided by at least a vague idea of how the world should be. Scientific work that focuses on *the world as it should be* is never entirely divorced from the world as it is. But that understanding has not resolved the (in) famous ‘is-ought problem’, the problem for which Friedrich and colleagues warn: “it is important to note that no empirical description of relationships between variables necessarily determines the values one *ought* to pursue” [2], emphasis in original.

Many public health research papers do exactly what Friedrich and colleagues warn for: they describe an empirical relationship between variables – for example, the correlation between availability of potable water and cases of diarrhea – and move directly towards an ‘ought’ statement – for example, that authorities ought to improve access to potable water. The underlying assumption is that authorities ought to do what it takes to reduce cases of diarrhea. Often, the underlying assumption is not controversial, and therefore it seems unnecessary to mention it. At times, however, the underlying assumption is controversial or insufficient. Using the same example as above, in places where potable water is very expensive to provide, and providing it may deplete scarce public resources that could be used more efficiently, it is not self-evident that authorities ought to provide potable water. And what if the majority of a community has access to private sources of potable water, and refuses to pay taxes to enable authorities providing water to the minority? Should democracy prevail in such a situation?

The (is-ought) problem that I want to highlight with these examples is that there is no empirical evidence for the underlying normative assumptions that are frequently used in public health. For Nozick, for example – who argues that “[n]o state more extensive than the minimal state can be justified” [3], page 297, and his minimal state is not responsible for the health or general wellbeing of its citizens, only for protection against violence, theft and fraud – the state has no responsibility to provide potable water, or healthcare, or education. One can argue that Nozick’s position is ethically *wrong*, or perhaps in conflict with national or international law, but it is quite impossible to *prove* empirically that his position is *false*.

### Global health: an interdisciplinary science, drawing on empirical and normative disciplines

If we accept the definition of global health proposed by Koplan and colleagues – namely: “global health is an area for study, research, and practice that places a priority on improving health and achieving equity in health for all people worldwide” [4] – then Sabine would probably have classified global health as a normative science: trying to state *what ought to be*. But global health relies heavily on empirical disciplines. As Koplan and colleagues argue: “many disciplines, such as the social and behavioural sciences, law, economics, history, engineering, biomedical and environmental sciences, and public policy can make great contributions to global health” [4].

Interdisciplinary scientific work comes with challenges. Biomedical sciences, law, and ethics do not have a common epistemology. A lawyer’s ‘truth’ – or ‘justified belief’ [5] – is of a different nature than a philosopher’s ‘truth’ or a physician’s ‘truth’. For a lawyer, it is true that high-income countries have a legal obligation to assist low-income countries in providing healthcare, because there is international law confirming that obligation, even in the absence of empirical evidence of high-income countries behaving as such. For some philosophers, high-income countries would have this obligation even in the absence of international law, because of the requirements of global justice. For most physicians, however, a ‘truth’ is a valid statement about something that is likely to occur under given circumstances – for example, patients with certain symptoms reacting to certain medicines in certain ways – and if states do not really behave as lawyers or philosophers argue they should, physicians will find it difficult to consider the corresponding normative statements as ‘truths’.

### To move from international to global health requires a new normative starting point

Public health (at the national level) can rely on the widely accepted assumption that “the national government is responsible for the health of the people”, as Rosen formulated it [6], page 444. Obviously, this normative statement leaves many normative questions unanswered – what level of resources can the national government reasonably mobilize; how should constrained resources be allocated? – but it provides a starting point. Furthermore, as long as global health was known (and practiced) as *international* health, or in the words of Koplan and colleagues, as “health work abroad, with a geographic focus on developing countries and often with a content of infectious and tropical diseases, water and sanitation, malnutrition, and maternal and child health” [4], the widely accepted normative starting point according to which “the national government is responsible for the health of the people” [6], page 444 was fit for purpose – the purpose being reducing health inequalities within ‘developing’ countries. But when *international* health becomes *global* health, and tries to identify and address global health inequities, the normative starting point of public health no longer works.

To be clear, the World Health Organization (WHO) defines ‘inequities in health’ as: “systematic and potentially remediable differences in one or more aspects of health across socially, economically, demographically, or geographically defined population groups or subgroups” [7], page 140. Whitehead adds that ‘inequity’ refers to “differences which are unnecessary and avoidable but, in addition, are also considered unfair and unjust” [8]. Thinking about ‘equity in health for all people worldwide’ therefore requires thinking about potential remedies for global health *inequalities*, and thinking about which of these remedies are required to advance justice and fairness: thus global health requires a normative starting point about what states ought to do across state borders. But there is no widely accepted answer to the question about what states ought to do to improve the health of people living in other states. Global health researchers who are focused on empirical research therefore need a dialogue with global health researchers who work on that normative question.

Likewise, global health researchers who focus on normative global health issues rely on empirical research. Even the most compelling legal or moral argument for identifying global health *inequalities* as global health *inequities* – as inequalities that are potentially remediable and unjust – requires guidance from empirical research on potential remedies. If, for example, there were no empirical evidence of international assistance improving the health of the people living in countries receiving international assistance, then the argument for international assistance – as a remedy against global health inequalities – would fail.

### Alternative normative starting points for international and global health

Lencucha offers four alternative “ethical positions” for foreign policy for health [9]:

- “Isolationism” – states have no responsibility whatsoever to assist other states in improving health;
- “Charity” – states can help other states, if they want, for as long as they want, and according to their own priorities;
- “Security” – states should help other states address health issues that are of common concern;
- “Cosmopolitanism” – humanity has a moral responsibility towards humanity, and therefore states should assist each other (or people should assist each other, across borders, using states as instruments).

Each of these ethical positions can be used as a normative starting point for international or global health, and they may lead to very different recommendations, regardless of what the empirical evidence tells.

Adopting ‘isolationism’ as the normative starting point for foreign policy for health would reduce the scope of global health to old school international health: recommendations should only consider the national situation with regards to needs, means and priorities. That could, for example, support the recommendation that low-income countries should prioritize HIV prevention over AIDS treatment [10], if it can be proven that HIV prevention is indeed more efficient than AIDS treatment and that many low-income countries cannot afford to do both. ‘Isolationism’ could also support the recommendation that low-income countries should focus their efforts to reduce maternal mortality on increasing skilled birth attendance, rather than on trying to increase skilled birth attendance and emergency obstetric care [11], if increasing skilled birth attendance is within the country’s means and emergency obstetric care is not.

Adopting ‘charity’ as the normative starting point for foreign policy for health may lead to a somewhat more generous form of international health. Again, recommendations should first and foremost consider the national situation with regards to needs, means and priorities. International assistance can help poorer countries, but such assistance would be a matter of charity, not a requirement of justice. (I would therefore argue that if one uses ‘charity’ as the normative starting point for foreign policy for health, one should not consider global *inequalities* in health as global *inequities* in health, because the potential remedy is not required by justice – that is why I think this normative starting point leads to a somewhat generous form of international health, rather than a form of global health.) On a more practical level: both AIDS treatment and emergency obstetric care in low-income countries could be recommended, as long as one has enough confidence in the depth of support of wealthier states for these issues (or rather not recommended, if one distrusts the reliability of charity-based aid).

If one adopts ‘security’ as the normative starting point for foreign policy for health, one arrives at a particular form of global health: not global health as defined by Koplan and colleagues, but global health as Feldbaum and Michaud describe it: “a means [used by countries] to improve security, project power and influence, improve their international image, or support other traditional foreign policy objectives” [12]. Under this form of global health, AIDS treatment would be recommended, because it would serve the interests of wealthier states to help poorer states control the epidemic as tightly as possible, while emergency obstetric care may not be recommended, because maternal mortality does not constitute a cross-border health security issue [13].

Under a ‘cosmopolitan’ approach to foreign policy for health – or a right to health approach, which represents a rather modest version of cosmopolitanism [14] – both AIDS treatment and emergency obstetric care would be recommended, because they save human lives, and wealthier countries ought to assist countries unable to provide these interventions.

There are alternative but similar grids of normative starting points: Frenk and colleagues’ description of an evolution in global health from “development aid” to “international cooperation” to “global solidarity” [15] corresponds with Lencucha’s ‘charity’, ‘security’ and ‘cosmopolitanism’. Brown’s “four normative approaches to global health” include “proximity”, “lifeboat ethics”, “utility”, and “cosmopolitanism” [16]. Stuckler and McKee’s “five metaphors about global-health policy” are essentially five different normative starting points for global health: “global health as foreign policy”, “global health as security”, “global health as charity”, “global health as investment”, and “global health as public health” [17].

In this paper, I will use Lencucha’s grid, because it is easy to understand yet sophisticated enough to serve my purpose: to illustrate that different normative starting points lead to different international or global health recommendations, while most international or global health recommendations that are based on empirical evidence do not clarify the normative starting point adopted by the authors. The other grids are mentioned to illustrate that there is no widely accepted grid of normative starting points, let alone a single widely accepted normative starting point, but also to illustrate a different part of the problem. Lencucha’s paper was published in *BMC International Health & Human Rights* – a medical journal – as a ‘debate’ paper, not a ‘research’ paper [9]. Frenk and colleagues’ paper [15] and Stuckler and McKee’s paper [17] were published in *The Lancet* – a medical journal – as a ‘viewpoint’ and a ‘comment’, not as ‘research’. Brown’s paper was published as a ‘research article’ in *Global Policy* [16] – *not* a medical journal. Opinion papers can be influential. But for most researchers with a background in empirical disciplines, conclusions reported in opinion pages do not carry the same weight as conclusions reported in research papers. ‘Truths’, for most physicians, are found in research papers.

### Dialogue

Several contemporary global health debates require a dialogue between global health researchers who are trained in empirical disciplines and global health researchers who are trained in normative disciplines. Lawyers and ethicists are not trained to assess the impact of different public health interventions and the inevitable uncertainties that emerge from empirical studies. They need physicians and epidemiologists to tell them what difference it makes to provide HIV prevention alone or prevention and AIDS treatment, or to provide skilled birth attendance alone or skilled birth attendance and emergency obstetric care. They need this information, for example, to be able to illustrate the difference between the ‘charity’, the ‘security’ and the ‘cosmopolitan’ approaches to foreign policy for health. But if researchers with a background in empirical disciplines omit some interventions from their research, because they adopt (implicitly) the ‘charity’ approach to foreign policy for health and prefer not to promote interventions that would rely on (unreliable) charity, then it becomes impossible for global health researchers with a background in normative disciplines to build their work on the conclusions derived from empirical evidence. For example, when Choulagai and colleagues conclude that “Nepal’s health system must develop strategies that generate demand for [skilled birth attendance] services”, without mentioning the option of providing emergency obstetric care [18], it is not easy for a human rights lawyer like me to understand why they did not consider emergency obstetric care. Is it because they are convinced that emergency obstetric care would not make a difference (would not reduce health inequalities)? Or is it because they are convinced that Nepal cannot afford to provide emergency obstetric care, and that the international assistance that could allow Nepal to provide emergency obstetric care will not be forthcoming?

Likewise, physicians and epidemiologists working in global health need lawyers and philosophers to clarify the consequences of the different normative starting points for global health. It is tempting to work within the status quo – to base recommendations on the assumption that the resources available today will also be the resources available tomorrow – but there is little empirical evidence for that assumption and it is ethically and legally problematic. According to Lencucha, the dominant normative starting point in global health policy is the ‘security’ approach [9], which provides a plausible explanation for the fact that we live in a world with a Global Fund to fight AIDS, Tuberculosis and Malaria, and no global fund to fight maternal mortality [19]. If global health recommendations are adjusted to that reality, and therefore more ambitious when it comes to fighting AIDS than when it comes to fighting maternal mortality, those recommendations tend to confirm the status quo, and reaffirm the dominant normative starting point.

However, if global health researchers with a background in empirical disciplines want to use a normative starting point that departs from the status quo, they will want a normative starting point that is based on rigorous research. If global health researchers with a background in normative disciplines want to provide guidance, they could present their findings in ways that help their colleagues to distinguish between merely personal opinion and professional opinion, the outcome of rigorous research.

## Discussion

What would it take to improve the dialogue between global health researchers who are trained in empirical disciplines and global health researchers who are trained in normative disciplines? In the background section, I argued that there are two sides to the problem:

- Global health researchers with a background in empirical disciplines seem reluctant to clarify the normative starting point they use;
- Global health researchers with a background in normative disciplines usually present their work in ways that do not help their colleagues with a background in empirical disciplines to distinguish between merely personal opinion and the results of rigorous research.

Before I try to explain why this is happening, and before I try to offer solutions, I should probably explain that I am a human rights lawyer, working at the Institute of Tropical Medicine in Antwerp (ITM), where most faculty members are physicians: I belong to one of the ‘sides’.

### Why are global health researchers with a background in empirical disciplines reluctant to clarify the normative starting point they use?

In their paper on “Evidence-based policy making in global health”, in the journal *Evidence-Based Medicine*, Yamey and Feachem use a graphic that shows how, over time, “global health policies based on evidence” are replacing “global health policies based on opinion or whim” [20]. This assessment will make most global health researchers with a background in normative disciplines frown. Are there any global health policy recommendations that are based on evidence, but not on personal opinion? Surely, if the evidence is strong, it must have influenced the opinion of the researchers who are using it. Probably, Yamey and Feachem wanted to distinguish ‘global health policy based on merely opinion or whim’ from ‘global health policy based on opinion based on evidence’. In any case, the dichotomy they use illustrates a conviction shared by many scientists who are trained in empirical disciplines: that there is something problematic about policy recommendations based on personal opinion.

The current trend of evidence-based global health builds on the earlier trend of evidence-based medicine or evidence-based public health [21], which tries to move away from public health “driven by crises, hot issues, and the concerns of organized interest groups” [22]. Who could disagree with moving away from the whims and concerns of organized interest groups as the driving forces of global health? But a normative starting point is essential for any global health policy recommendation, and normative starting points cannot be derived directly from empirical evidence, because of the ‘is-ought problem’ discussed above. Normative starting points can be more than merely personal opinions (or whims): they can be found in law or ethical principles and theories, or derived from these through logical reasoning, but they cannot be empirically proven. In trying to exclude the whims and concerns of organized pressure groups from global health policy making, and lumping well-reasoned normative opinions together with whims, the trend of evidence-based global health has discredited the explicit use of normative starting points that are at the heart of global health.

An alternative or complementary explanation is that present global health policies are very different from the ones that are required by the normative starting point that most global health researchers support. Returning to Lencucha’s grid [9], ‘isolationism’ is contradictory to the very idea of global health. Not many global health researchers support ‘charity’ as the appropriate normative starting point for global health: even if the use of expressions like ‘donors and recipients’ or ‘sustainability’ (narrowly defined as ‘capacity to endure without external assistance’) reflects an acceptance of the reality that much international assistance is, at present, essentially charity, that does not mean that the researchers who use these expressions agree that global health ought to be based on charity. Likewise, references to common interests or advantages expected to result from improved global health – economic growth in developing countries from which all countries would benefit, infectious disease control, or other ‘global public goods’ – seem to be used more often by global health researches who think these arguments will appeal to politicians, not by global health researchers who agree that these arguments ought to be the driving forces of global health. Most global health researchers probably favor global health based on ‘cosmopolitanism’. But a cosmopolitan approach to global health is far more demanding than the global health policies we have at present. This creates a dilemma for many global health researchers: should they recommend policies based on the normative starting point they support themselves (together with the empirical evidence they found), knowing that these recommendations will probably not become reality, because global health policy makers use a different normative starting point, or should they recommended policies that are far below the standard they would consider as appropriate themselves, but still better than present policies, and more likely to be implemented? Some global health researchers avoid this dilemma by formulating two options, like Costello and colleagues do when they argue that “[g]overnments need to be held accountable for the comprehensive provision of facility-based midwifery and obstetrical care, which should be a key component of any national safer motherhood strategy”, yet add that “[i]t is naive, though, to assume that this strategy is realistic in all contexts for the next decade”, and conclude by recommending improved skilled birth attendance (not necessarily facility-based, and therefore not necessarily including emergency obstetric care) as the non-ideal but feasible policy [11]. A two-tier recommendation like this, however, is rather unusual, because researchers are understandably reluctant about ‘undermining’ the recommendation they are making by admitting they would personally support something else, something better.

### Why are global health researchers with a background in normative disciplines not presenting their work in ways that help their colleagues with a background in empirical disciplines distinguish between merely personal opinion and the results of rigorous research?

There is no dearth of rigorous research into the normative foundations of global health. In recent years, the normative foundations of global health have been explored in monographs by lawyers (Gostin [23], Tobin [24], Murphy [25]) and philosophers (Wolff [26], Venkatapuram [27]), and in many papers and book chapters. But it is hard to find a paper on the issue that has been published as ‘original research’ in a medical journal. Why?

First, and perhaps foremost, most researchers with a background in normative disciplines would probably question the dichotomy between ‘personal opinion’ and ‘results of rigorous research’. When rigorous research yields given outcomes, those outcomes will influence the opinion of the researcher. Normative researchers may dislike the outcome of some of their research – for example, that international intellectual property law forces governments to pay prices for some medicines that these governments can hardly afford to pay – but their finding that there is something problematic about international intellectual property law becomes part of their opinion too. Lawyers have a solution for this tension: if necessary they distinguish between ‘lex lata’ opinions and ‘lex ferenda’ opinions – ‘lex lata’ opinions are in accordance with present law, ‘lex ferenda’ opinions are in accordance with the law as they think it should be. They then have a two-tier opinion, based on their research: according to the law, something ought to happen; according to certain principles, something else ought to happen (and the law ought to be changed). Furthermore, researchers with a background in normative disciplines accept that no normative statement can be based on empirical evidence only. For researchers with a background in normative disciplines, what matters is the difference between properly and improperly researched and argued personal opinions.

Second, there is the issue of the length of papers medical journals accept as research papers. To give a few examples: *The Lancet* advises authors to keep research papers below 3,000 words; for *Human Rights Quarterly*, papers should average between 5,000 and 10,000 words in length, and the editors of *Ethics* prefer original research articles to be between 5,000 and 12,000 words. This is not because lawyers and philosophers like to write long papers, but because it is difficult to summarize normative research in less than 5,000 words. Whereas the outcomes of a randomized controlled trial can be summarized in a table – containing symbols like degrees of fever, weeks of treatment, positive or negative for outcomes – the ‘findings’ used in normative research – legal texts, ethical principles – have to be spelled out and explained carefully if the researcher wants to avoid distorting them. If ‘fever’ means more or less the same thing for most people, ‘fairness’ does not. When global health researchers with a background in normative disciplines try to write a paper of less than 3,000 words, it will often be a paper in which they only develop the arguments that support their personal opinion, because there is no space for arguments supporting an alternative opinion – and it will look like an improperly researched and argued personal opinion.

Third, there is the issue related to the structure of papers. Since the 1980s, the ‘introduction, methods, results, and discussion’ (IMRAD) structure is “the only pattern adopted in original papers” in leading medical journals [28]. In Wu’s words:

- In the *Background* section, the researcher should answer the question “Why did you do it in the first place?”
- In the *Methods* section, the researcher should answer the question “How did you do it exactly?”
- In the *Results* section, the researcher should answer the question “What did you find?”
- In the *Discussion* section, the researcher should answer the question “What does it mean after all and so what?” [29].

For normative research, it makes little sense to disentangle these questions. It is difficult to explain why one investigated a normative issue without explaining the meaning of the issue; the common method is logic and sound reasoning and it is applied throughout the research, and ‘results’ will only be considered as such if they are meaningful. Therefore the same arguments have to be developed – and repeated – in several sections, which would make the paper even longer.

Last but not least, when global health researchers with a background in normative sciences try to conform to medical journal standards – the length, the IMRAD structure – they will often find that their paper is refused as a research paper but accepted as an opinion paper. This has happened to me and my colleagues numerous times. For a paper that was recently published by *BMC International Health and Human Rights*, we used the IMRAD structure and tried to keep the length below 4,000 words – provoking a comment by one of the reviewers, a philosopher, that is was a very short paper – but it was refused as a research paper and accepted as an opinion paper [30]. The editors argued that they did not have a standard allowing them to distinguish this paper from a paper that represents (merely) the personal opinion of the authors.

### Can global health researchers with a background in empirical disciplines be encouraged to clarify the normative starting point they use?

As long as global health policies are divided into “global health policies based on evidence” and “global health policies based on opinion or whim” [20], it will be difficult to convince global health researchers with a background in empirical disciplines to be open about the normative starting point they use. To some extent, normative starting points are inevitably based on personal opinion, because they cannot be empirically proven, and as long as ‘evidence-based global health’ ranks personal opinion together with ‘whim’, global health researchers will refrain from explaining to what extent their recommendations are based on their personal opinion with regards to the normative starting point for global health.

However, a paper Hammonds and I wrote recently for *Tropical Medicine and International Health*, on “Right to health and global public health research” [19], seems to have sparked a rather healthy – in my opinion – debate among physicians (mostly) on the “Politics in Global Health Policy” in the *International Journal of Health Policy and Management*. Bruen and Brugha argue that global health policies “are never politically neutral” [31]; McCoy and Singh write that “[i]t seems extraordinary that we need reminding of the fact that policy-making is fundamentally political; and that there is no such thing as a ‘neutral policy’” [32]; and Harmer argues: “To research health policy is to research politics” [33]. Researching politics without researching the normative starting points that underpin politics makes no sense. I sincerely hope that debates like these will encourage global health researchers with a background in empirical disciplines to clarify the normative starting point they are using.

I anticipate, however, that some of the global health researchers with a background in empirical disciplines will argue that there is an important difference between *describing* the politics that underpin global health at present, and expressing a normative opinion about those politics. Some of them will argue that it is merely their job to describe, not to prescribe. I would agree, but only to a very limited extent: only for global health research that remains empirical from the beginning to the end – i.e., research that does not lead to any recommendation. As soon as research serves as the basis for a recommendation, it should *evaluate* – i.e., to assess the value of – the politics on which it is based, otherwise it confirms those politics implicitly. As discussed above, using Lencucha’s grid again [9], if one assumes that ‘security’ considerations drive foreign policy for health (while believing that ‘cosmopolitan’ considerations ought to drive foreign policy for health), and one therefore recommends global health policy based on the assumptions that there will not be enough reliable international assistance for more ambitious maternal health policies (while there may be enough reliable international assistance for more ambitious infectious disease control policies) one will end up recommending a combination of global health policies driven by ‘security’ considerations. Does it mean then that all global health research should be based on an ‘appeal to faith’ – a belief that a better world is possible – instead of an ‘appeal to tradition’, as Bruen and Brugha [31] summarized Hammonds’ and my position [19]? There is a truism that ‘ought’ implies ‘can’ and it means that one should not recommend policies that are not possible in the real world. If one does not believe that global health policies driven by ‘cosmopolitan’ considerations are possible, one should indeed not recommend them, but then one should state that one proposes global health policy based on ‘security’ or perhaps ‘charity’ as the best possible global health policy in the real world – allowing colleagues to understand how the recommendations are colored by that normative starting point.

To be sure, I am not asking all global health recommendations to begin or end with extensive elaborations of the normative starting point used by the researchers. A brief reference to where one stands with regards to Lencucha’s [9], Frenk and colleagues’ [15], Brown’s [16] or Stuckler and McKee’s [17] grid would be sufficient to inform colleagues. Furthermore, as I explained above, I suspect that the politics that underpin global health policy at present are very distant from the normative starting point that most global health researchers support, which creates a tension between ‘policies for the real world’ and ‘policies for the better world’. Here again, a simple acknowledgement of alternative ‘policies for the better world’ would suffice. I shall illustrate this, using a realm other than global health policy. I respect policy recommendations that try to eliminate *hazardous* child labor, and that are based on the assumption that eliminating *all* child labor is not possible in the short run, but think it is essential to mention that eliminating all child labor remains the ultimate goal. Likewise, I appreciate Costello and colleagues’ comment that “[g]overnments need to be held accountable for the comprehensive provision of facility-based midwifery and obstetrical care” and their acknowledgment that their recommendations are based on the assumption that this strategy is *not* “realistic in all contexts for the next decade” [11]: that allows me, a human right lawyer working in global health, to understand that their recommendations are not ambitious enough to realize the right to health. It also allows me to understand that the “Technical guidance on the application of a human rights-based approach to the implementation of policies and programmes to reduce preventable maternal morbidity and mortality” issued by Office of the United Nations High Commissioner for Human Rights [34] – containing more ambitious recommendations than the ones formulated by Costello and colleagues – are based on a different normative starting point, not necessarily on different empirical evidence. It would be extremely useful if all global health researchers who are trained in empirical disciplines would offer similar clarifications.

### Can global health researchers with a background in normative disciplines be encouraged to present their work in ways that help their colleagues with a background in empirical disciplines to distinguish between (merely) personal opinions and the results of rigorous research?

I suspect that many of my colleague global health researchers with a background in normative disciplines would like to engage in a dialogue with global health researchers with a background in empirical disciplines. But some of them will argue that if the interdisciplinary nature of global health were well understood and respected by all global health researchers – allowing researchers from different disciplinary backgrounds to work within their own paradigm [35] – they would not have to adapt to the expectations of their colleagues with a background in empirical disciplines. The latter should accept that if a paper is published as a research paper in a peer-reviewed legal or philosophical journal, it is the result of rigorous research, not merely personal opinion (or whim).

However, many of us have come to realize that in spite of all the rhetorical support for interdisciplinary global health research, it remains a challenge to cross the boundaries between normative and empirical disciplines. When our colleagues with a background in empirical disciplines look for knowledge – for ‘truths’ they can work with, that can serve as a building block for additional research – they usually look in research papers in medical journals.

When I discuss this with colleagues at the ITM, it reminds them of the ‘wall’ that existed – and still exists, to some extent – between quantitative and qualitative research. Qualitative researchers felt or feel that “the rigid requirements (i.e., word count) of medical journals and reviewers’ attempts to “quanti-sise” qualitative research (apply assumptions of the quantitative paradigm to quality assessment) prohibit publication of their work” [36]. Likewise, some global health researchers with a background in normative disciplines feel pressure to ‘empiricalize’ their work, in order for it to be considered as ‘real’ research: the empirical findings of interviews with experts about the meaning of the right to health were published as a research paper in this journal, *BMC International Health and Human Rights* [37], the results of comparing the texts of different norms were published as correspondence [30]. I do not want to argue that empirical research is useless for normative issues, but one has to understand the limitations. For example, interviewing hundreds of taxpayers of countries providing international assistance about their global health priorities – giving them the choice between fighting Ebola through a ‘vertical’ program or supporting health systems in low-income countries so they would be able to control Ebola outbreaks – would provide useful insights into the politics that drive global health policy but not the answer the question about the politics that ought to drive global health policy.

The solution identified for blending qualitative with quantitative research may also improve the dialogue between empirical and normative disciplines. This journal, like many other medical journals, uses Clark’s guidelines for reviewing qualitative research manuscripts [36]. These standards are known by the acronym RATS, which stands for:

- Relevance of study question;
- Appropriateness of qualitative method;
- Transparency of procedures;
- Soundness of interpretive approach.

I think *RATS* could also be used as guidance for reviews of papers reporting normative research – or normative inquiries – even by reviewers who do not have a background in normative disciplines.

The *relevance* of a normative inquiry can be judged by assessing the relevance of the global health policy issue at stake, and by assessing whether different normative starting points lie at the roots of different recommendations. For example, if some global health researchers argue that in some circumstances public health agents should focus on cancer prevention, while others recommend providing cancer treatment, then doubts about the effectiveness of cancer treatment – based on inconclusive empirical evidence – could lie at the root of the different recommendations, and then a normative inquiry may again seem rather irrelevant (empirical studies examining the effectiveness of cancer treatment in different circumstances would be more urgent). However, if recommendations differ because researchers appear to use different normative starting points – about national and international responsibilities for providing cancer treatment – a normative inquiry would be relevant.

The *appropriateness* of a normative inquiry can be judged by the selection and the justification of the arguments that are considered and compared. Are the arguments relevant for the issue? Have all the relevant arguments been considered and justified?

The *transparency* of a normative inquiry can be judged by the criteria researchers used to consider or omit arguments, and whether these criteria are explicitly mentioned. For example, if a normative inquiry considers only arguments developed by philosophers known as ‘cosmopolitan’ thinkers, omits the arguments of philosophers who favor more state-centered interpretations of global justice, and does not explain why the arguments of philosophers who favor more state-centered interpretations of global justice were not considered, the results are probably misleading the readers.

Finally, even a normative inquiry that is relevant, appropriate and transparent, should still be assessed for its *interpretative soundness*. For example, a normative inquiry that focuses on national and global disparities in under five mortality [38], may lead to the conclusion that both the national and the global inequalities observed should be considered as *inequities*, i.e. inequalities that are remediable, unfair and unjust. But if it were followed directly by a recommendation that all states of the world pool all their public resources for health, to allocate them in the most cost-effective way to maximize the health of humanity, that would – in my opinion – lack interpretative soundness. Such a recommendation could serve as a light house for incremental changes, but not as a foundation for global health policy in the immediate future.

I am aware that the summary transposition of *RATS* guidelines to normative research that I am offering here does not do justice to their richness. Any attempt to formulate comprehensive *RATS*-like guidelines for normative research would, at present, be haughty. Clark developed *RATS* using existing guidelines for preparing qualitative research projects, critical evaluations of qualitative research papers done by others, and her own professional experience [36]. I think that a consultation involving global health researchers from different disciplines and editors of medical journals would be required to develop *RATS*-like guidelines, appropriate for normative research. The global health dialogue I have in mind needs to start with a dialogue about what it takes to understand each other.

Finally, I am not arguing that all papers or books about normative issues that are relevant for global health policy ought to be written in line with *RATS*-like guidelines. The above-mentioned argument about the interdisciplinary nature of global health research – which should allow researchers from different disciplines to work and report within their own paradigm – remains valid, in my opinion. I am only proposing a solution for global health researchers with a background in normative disciplines who want to make it easier for their colleagues with a background in empirical disciplines to see the difference between (merely) personal opinions and the results of rigorous research.

## Summary

If global health research wants to achieve the goal of “achieving equity in health for all people worldwide” [2], it needs to build on ‘truths’ from empirical and normative disciplines, and these are ‘truths’ of different epistemological natures. If only to explain what ‘equity in health for all people worldwide’ means, a normative starting point is needed.

A single common paradigm for all disciplines that contribute to global health may not be possible, perhaps not even desirable at present. The gap between present realities in global health policy and the normative starting position that most global health researchers support is such that an attempt to bridge it could lead to either unrealistic or unfair policy recommendations. However, a better dialogue between global health researchers with a background in empirical disciplines and global health researchers with a background in normative disciplines could help to identify incremental steps towards ‘equity in health for all people worldwide’ – towards *global health*.

Global health researchers with a background in empirical sciences could relatively easily declare the normative starting point on which their recommendations are built, and explain whether they personally agree with that starting point or if they rather reluctantly accept it as the current reality. That would allow global health researchers with a background in normative sciences to distinguish between a recommendation that is based on the acceptance of the present politics of global health and a recommendation that represents what the researchers who formulated the recommendation truly believe to be the right recommendation – the one that ought to be implemented to achieve ‘equity in health for all people worldwide’.

Global health researchers with a background in normative disciplines could present their findings in ways that would help global health researchers with a background in empirical disciplines to distinguish between what is merely a personal opinion and what is the result of rigorous normative research. Guidelines that are similar to the *RATS* guidelines, developed to review qualitative research, could be adapted for reporting normative inquiries. Achieving this will require a consultation involving global health researchers from different disciplines and editors of medical journals.

## Abbreviations

IMRAD: Introduction, Methods, Results, Discussion
ITM: Institute of Tropical Medicine
RATS: Relevance, appropriateness, transparency, soundness
WHO: World Health Organization
